# Injectable Hydrogels Based on Cyclodextrin/Cholesterol Inclusion Complexation and Loaded with 5-Fluorouracil/Methotrexate for Breast Cancer Treatment

**DOI:** 10.3390/gels9040326

**Published:** 2023-04-12

**Authors:** Saud Almawash, Ahmed M. Mohammed, Mohamed A. El Hamd, Shaaban K. Osman

**Affiliations:** 1 Department of Pharmaceutical Sciences, College of Pharmacy, Shaqra University, Shaqraa 11961, Saudi Arabia; salmawash@su.edu.sa (S.A.);; 2 Department of Pharmaceutics and Pharmaceutical Technology, Faculty of Pharmacy, Al-Azhar University, Assiut 71524, Egypt; 3 Department of Pharmaceutical Analytical Chemistry, Faculty of Pharmacy, South Valley University, Qena 83523, Egypt

**Keywords:** β-cyclodextrin polymers, 5-fluorouracil, methotrexate, cholesterol, polyethylene glycol, injectable hydrogels, histopathology, MTT assay, breast cancer

## Abstract

Breast cancer is the second most common cancer in women worldwide. Long-term treatment with conventional chemotherapy may result in severe systemic side effects. Therefore, the localized delivery of chemotherapy helps to overcome such a problem. In this article, self-assembling hydrogels were constructed via inclusion complexation between host β-cyclodextrin polymers (8armPEG20k-CD and pβ-CD) and the guest polymers 8-armed poly(ethylene glycol) capped either with cholesterol (8armPEG20k-chol) or adamantane (8armPEG20k-Ad) and were loaded with 5-fluorouracil (5-FU) and methotrexate (MTX). The prepared hydrogels were characterized by SEM and rheological behaviors. The in vitro release of 5-FU and MTX was studied. The cytotoxicity of our modified systems was investigated against breast tumor cells (MCF-7) using an MTT assay. Additionally, the histopathological changes in breast tissues were monitored before and after their intratumor injection. The results of rheological characterization indicated the viscoelastic behavior in all cases except for 8armPEG-Ad. In vitro release results showed a variable range of release profiles from 6 to 21 days, depending on the hydrogel composition. MTT findings indicated the inhibition ability of our systems against the viability of cancer cells depending on the kind and concentration of the hydrogel and the incubation period. Moreover, the results of histopathology showed the improvement of cancer manifestation (swelling and inflammation) after intratumor injection of loaded hydrogel systems. In conclusion, the obtained results indicated the applicability of the modified hydrogels as injectable vehicles for both loading and controlled release of anticancer therapies.

## 1. Introduction

The design of injectable hydrogels attracted the researchers’ attention because of their suitability as targeted, controlled, and sustained pharmaceutical delivery devices to the site of the disease. Consequently, the expected adverse effects of systemic exposure to such pharmaceutics can be diminished [1]. Additionally, the reduction in adverse effects on localized parenteral administration of such hydrogels is due to their ability to overcome the poor solubility of pharmaceutics. This causes the required therapeutic doses to be lowered, and an acceptable amount can reach the site of action [2,3]. Due to the existence of hydrophobic cavities in the structure of β-cyclodextrin (β-CD), it can engulf a hydrophobic molecule (guest) and form ahost–guest inclusion complex. However, the native β-CD cannot form a stable self-assembling hydrogel due to its low molecular weight and poor aqueous solubility. The polymerization of β-CD (pβ-CD) with epichlorohydrin (EP), in certain conditions, can overcome such shortening. Therefore, several physical hydrogel systems have been constructed from the inclusion complexation between pβ-CD with different hydrophobic molecules and the capping of high-molecular-weight polymers [4,5,6]. The pβ-CD is utilized instead of free β-CD molecules (poorly soluble and uninjectable compounds) to ensure the formation of more stable, injectable, and biocompatible hydrogel systems [7].

According to recent WHO reports, cancer is the second leading cause of death worldwide, causing around 10 million deaths in 2020 globally [8,9]. Depending on the type and status of cancer, treatment approaches, such as surgery, radiation, chemotherapy, hormonal therapy, immunotherapy, and gene therapy, may be chosen [10]. Chemotherapy, using a cytotoxic agent, remains the most predominant approach for cancer management and recurrence inhibition [11,12]. In this context, 5-fluorouracil (5-FU) is an antineoplastic antimetabolite agent which has been utilized in the management of different kinds of cancer, including ovarian, breast, liver, lung, and skin cancers [13,14,15]. Nowadays, traditional single chemotherapy has been replaced by different combinational regimens, which use multiple drugs with different mechanisms to obtain a synergistic antiproliferative effect and overcome individual high-dose resistance [16].

Notably, it was previously stated that administering methotrexate (MTX) together with 5-FU for the same cases enhances 5-FU uptake into normal and defective tissues [17]. However, administering the individual therapies orally or parentally exposes normal tissues to drug-induced toxicity due to their lack of selectivity [18,19]. Therefore, the attention of most pharmaceutical researchers was directed toward targeting or localizing anticancer therapies [20]. As a result, various sustained-release injectable systems were designed, such as liposomes [21], microemulsions [22], nano- and microparticles [23,24], micelles [25], implants [26], and self-assembling hydrogels [7,27,28,29], for this purpose.

In the present study, we assembled different hydrogel systems based on the inclusion complexation of host and guest molecules. We selected two host polymers of β-CD (pβ-CD and 8armPEG-CD) and two guest polymers (8armPEG-chol and 8armPEG-Ad). The modified hydrogel systems were utilized for the loading and release of two different anticancer drugs, named 5-FU and MTX. Then, the constructed hydrogels were characterized by their clarity, pH, drug content, fluidity, homogeneity, and in vitro release. Additionally, the rheological properties of the modified hydrogels were investigated by measuring the frequency, stress, and temperature sweep. In addition, the morphological features of the final hydrogel were examined by SEM. Furthermore, a kinetic model for the in vitro release behavior was investigated providing insight into the mechanism of drug release from different hydrogel systems. The injectability of the modified hydrogels was investigated. Moreover, the cytotoxicity of our modified systems was studied against breast cancer cell membranes (MCF-7) using an MTT assay. In addition, the histopathological changes in breast tissues were monitored before and after the intratumor injection of drugs. Altogether, this pharmaceutical work will provide deeper insights into the utility of a safe injectable device for localized and sustained release of the selected anticancer therapies for breast cancer management.

## 2. Results and Discussion

### 2.1. Synthesis and Characterization of the Polymers Utilized for Hydrogels Formation

The EP (cross-linking agent) was successfully utilized in the synthesis of water-soluble linear pβ-CD in an alkaline medium. The obtained pβ-CD showed 65% substitution degree and 96 kDa molecular weight. In addition, the grafted 8armPEG20k-CD was synthesized from the reaction of 8armPEG-amines with β-CD-CHO via reductive amination in a strongly alkaline medium. The synthesized polymer was obtained with a 90% yield and 97% degree of substitution (Table 1). Moreover, 8armPEG20k-chol was successfully synthesized and collected with about 85% yield and 75% degree of substitution. Regarding the 8armPEG20k-Ad, the product was obtained successfully with 90% yield and 95% substitution (Table 1).

### 2.2. Hydrogels’ Preparation and Drug Loading

**Figure 1:**
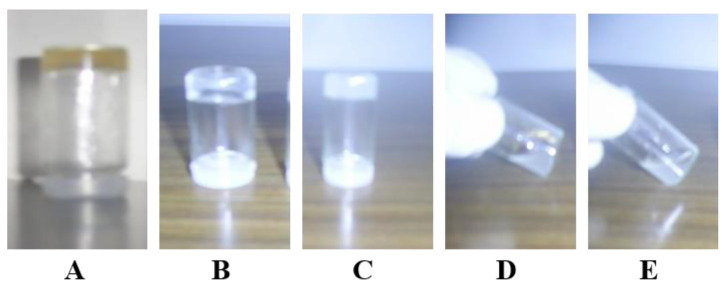
Photos of self-assembling hydrogel based on CD-chol/Ad inclusion complexation: (**A**) 8armPEG20k-chol/8armPEG20k-CD, (**B**) 8armPEG20k-Ad/pβ-CD, (**C**) 8armPEG20k-chol/pβ-CD, (**D**) 8armPEG-OH/pβ-CD, and (**E**) 8armPEG20k-chol/native β-CD.

Different hydrogel systems were formed by rehydration of a lyophilized mixture of the host and guest polymers. Figure 1 showed the chol/CD hydrogel networks of different formulas, 8armPEG20k-chol/8armPEG20k-CD (formula A), 8armPEG20k-Ad/pβ-CD (formula B), and 8armPEG20k-chol/pβ-CD (formula C). In contrast, both formulas formed from 8armPEG20k-OH/pβ-CD (formula D) or 8armPEG20k-chol/native β-CD (formula E) did not show hydrogel networks and behaved just as a viscous liquid. Regarding the loading of the drug mixture, the calculated amounts of 5-FU and MTX were dissolved into PBS (the rehydrating solution). This loading technique played a key role in controlling the release of the drugs as the loading capacity of the drug by such technique increases the drug’s affinity to the hydrogel base, containing a high concentration of the free CD cavities that incorporate the drug and, consequently, improve its solubility on the one hand with a controlled drug-releasing behavior, on the other hand [30].

### 2.3. Characterization of the Prepared Hydrogels

- Visual appearance and pH of the prepared hydrogels

All modified hydrogel systems were subjected to visual inspection by the naked eye for their organoleptic properties. The results indicated that all the investigated systems were uniform and smooth with no phase separation or lumps. Regarding the color of the modified gel systems, the results, illustrated in Figure 1, showed that the hydrogel system A (8armPEG20k-chol /8armPEG20k-CD) has a pale-yellow color. While in the cases of both hydrogel systems B and C which are composed of 8armPEG20k-chol /pβ-CD and 8armPEG20k-Ad /pβ-CD, respectively, the color was white and turbid. Moreover, it was observed by the naked eye that systems D and E had no 3D structure (i.e., no gel formation was obtained) at the same concentration.

Regarding the pH measurement, the results showed that the values of pH of the modified hydrogels ranged from 7.2 to 7.4, as listed in Table 2. The obtained data indicated the suitability of all gel formulations for subcutaneous injection. This is in agreement with the fact that the pH of any injectable dosage form should be in the range of 6.5–7.4 to ensure compatibility and safety and to avoid irritation or damage to human tissues [31,32].

- Rheological properties

To investigate the rheological properties of the modified system and to realize if they are real gels or just viscous constructs. In this regard, there are very important rheological parameters, including loss modulus (G″), storage modulus (G′), gel strength (G*), and viscosity, which were investigated as a function of temperature, frequency, and shear stress. The results, displayed in Table 2, showed that systems A and C showed a viscoelastic behavior since the values of G′ were higher than those of G″. In contrast, system B, composed of 8armPEG20k-Ad/pβ-CD, exhibited a viscous behavior since G′ ˂ G″, although it looks like gel as illustrated in Figure 1. This finding indicates that the obtained mixture is just a viscous construct. 

Regarding the viscosity data, formula C showed the highest viscosity value compared to the other formulas. The obtained results may be attributed to the higher density of the cross-linking as the higher molecular weight of pβ-CD compared to 8armPEG20k-CD. In addition, the higher concentration of hydrogel powder was accompanied by higher viscosity for the same reason. The obtained results and interpretation are in good agreement with previously published data [33].

As for the thermo-reversibility of the modified systems, it was noted that the panel of G′ values decreased stepwise with the increase in the G″ panel until the two panels cross each other. The point of cross-over is called transition temperature T_gel_ after this point, the inversion of two panels occurred since the G″ will be higher than G′, indicating gel to sol transition. Interestingly, the re-cooling caused G′ to be higher than G″ to re-cross each other (cross-over point or T_gel_) (Table 2) converting the viscous liquid into a viscoelastic behavior.

The values of T_gel_ were 42 °C and 68 °C for formulas A and C, respectively. In contrast, formula B showed no temperature cross-over point due to the panels of both G′ and G″ values being parallel to each other and G′ < G″ all over the whole measurement, indicating that system B behaves just as viscous liquid. Both the strength (G*) and the stability (resistance to be destroyed) of the different constructed hydrogel systems were investigated by stress sweep measurements. The results revealed that the value of G* varies significantly (*p* ≤ 0.05) depending on the composition of the modified hydrogel systems. The G* value of formula C (10% *w*/*v* 8armPEG20k-chol/pβ-CD) was higher than that of formula A (30% *w*/*w* 8armPEG20k-chol/8armPEG20k-CD). The results were attributed to the higher molecular weight of pβ-CD (96 kDa) compared to that of 8armPEG20k-CD (28 kDa), which, consequently, forms more cross-linking points of inclusion complex between CD cavities and cholesterol moieties.

In addition, the higher concentration of CD molecules played a vital role in the destruction of the micelles, formed from the self-aggregation of cholesterol, and hence improves the stability of the constructed hydrogel networks. Moreover, it has been noted that the value of (G*) was higher in the case of a higher solid concentration of the same hydrogel system for the same reason. A similar finding was observed regarding the values of the gel stability (Table 2).

The oscillatory frequency sweep experiments were performed at a frequency range of 0.1 to 10 Hz. In all cases, the rheograms started with viscous behavior since the values of G″ > G′. After a certain frequency point (cross-over point), both two panels crossed each other. After a further increase in frequency, the value s of G′ will be higher than those of G″ indicating the viscoelastic behavior (gel formation). The cross-over frequency points were recorded for all the constructed hydrogels systems and the data were listed in Table 2. The low values of the cross-over point indicate the fast formation of the hydrogels. Our results showed that formula C had a higher value of cross-over point compared to formula A. This finding confirmed the above-mentioned results regarding both the stress and temperature sweep experiments. In contrast, formula B did not show any cross-over point because the values of G″ ˃ G′ for the whole rheogram, which indicates the viscous behaviors of the system. This finding and interpretation were in agreement with previously reported data, where PEG-chol exhibits a higher affinity to β-CD when compared with PEG-Ad [34].

- SEM Morphological Studies

**Figure 2:**
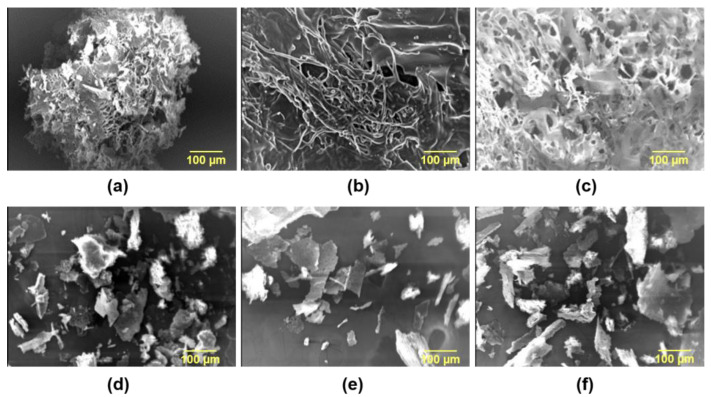
SEM micrographs showing the channels and cross sections of different lyophilized hydrogel systems at a magnification power (×100). (**a**–**c**) The graphs display the hydrogel formulas: (**a**) formula A, (**b**) formula B, and (**c**) formula C. (**d**–**f**) The graphs display the physical mixtures of the hydrogel components: (**d**) physical mixture of formula A, (**e**) physical mixture of formula B, and (**f**) physical mixture of formula C.

The examination of the surface morphology of hydrogels, intended for such mixture delivery applications, was very indicative due to the degree of porosity, which plays a vital role in determining the hydrogel properties, such as drug loading capacity, drug release power, hydrogel swelling, and water uptake [35,36]. Figure 2 shows the SEM micrographs of all constructed hydrogel systems compared to their corresponding physical mixtures.

The results showed that the hydrogels had porous structures which are apparent at magnifications power (×100). The porous structures of pβ-CD/8armPEG20k-chol hydrogel systems were interpreted into a formation of the inclusion complexes (cross/links) between host and guest molecules. These results and interpretations were in good agreement with other reports [36,37,38,39,40]. In contrast, the physical mixtures for all the formulas at the same magnification power (×100) showed no signs of porosity or cross-linking, where distributed powder blocks have been observed.

### 2.4. The In Vitro Release Studies

Firstly, on scanning all the ingredients which were used in the construction of the present hydrogels, it was found an interference at their specific wavelengths of λ_max_ 266 nm of 5-FU and 302 nm of MTX [7,41]. As a result, the measurement of both 5-FU and MTX in a mixture had an overlapped peak. A new derivative UV-spectrophotometric method was developed and validated by the authors [42,43] for drug quantification.

**Figure 3:**
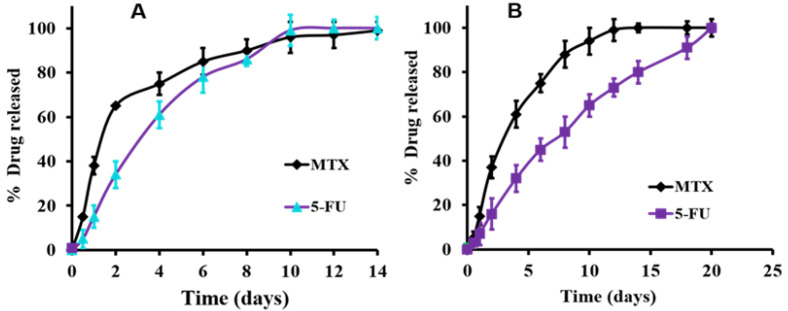
In vitro release profiles of both 5-FU and MTX from (**A**) hydrogel formula A, composed of 30%, *w*/*v* 8armPEG-chol/8rmPEG-CD (1:1%, *w*/*w* ratio), and from (**B**) hydrogel formula C, composed of 10%, *w*/*v* 8armPEG20k-chol/pβ-CD (1:1%, *w*/*w* ratio) at 37 °C in PBS.

To make sure that the whole drug mixture was released and there are no destructions or engagements of any of them by the constructed hydrogel system, the release experiment was allowed to continue until the complete release of the loaded drug mixture. Figure 3A showed the release profiles of the 5-FU/MTX mixture from the formula A, constructed from 30% *w*/*v* 8armPEG-chol/8armPEG-CD (equal *w*/*w* ratio) in PBS. The results exhibited that the release rate in the case of MTX was higher (about 100% of MTX released within the first 2 days) than the release rate of 5-FU (100% release was achieved within 8 days). Regarding formula C (illustrated in Figure 3B), similar results were collected with the extension of the release, where 100% MTX and 5-FU were released within 6 days and 14 days, respectively. Fortunately, these results support our target goal of co-administration of MTX with 5-FU for a synergistic effect. Noteworthy, owing to the higher solubility of MTX compared to 5-FU [44], the present study was intended to adjust the initial release of MTX before that of 5-FU. Eventually, this will help improve the synergistic effect of MTX and 5-FU.

According to the above-mentioned results, the kind of hydrogel formula played a vital role in the drug release profiles. In this regard, the fastest release of 5-FU was achieved in the case of formula A and followed by formula C. This finding may be attributed to the lower viscosity and gel strength of the hydrogel system (A), compared with the hydrogel system C (data presented in Table 2 and Figure 3B). Importantly, formula B which is composed of 8armPEG-Ad/pβ-CD was degraded and dissolved totally within 1 h of the release study. This result may be attributed to system B which behaves as a viscous liquid and not as a real gel, as indicated by the rheological studies. These results confirmed the utility of such modified systems for localized, extended, and controlled release of the selected anticancer drugs, which are the main goals of our current study.

### 2.5. Kinetic Model Studies

The release mechanism of the loaded anticancer drugs (5-FU and MTX) was detected differently by plotting the release date against time according to the equations of different kinetic orders, as mentioned in the experimental part. The curve exhibiting the highest R-value will be considered the best-fitted model, which represents the actual drug release mechanism.

Table 3 presents the release kinetics of 5-FU from different selected hydrogel systems. It was observed that the release mechanism of 5-FU followed the Higuchi diffusion model in the case of formula A. However, in the case of the Formula C, the suitable model was a zero-order model. A similar finding was obtained in the case of the release mechanism of MTX. The obtained results may be owed to the law density of the formed gel networks in cases of formula A which make both the investigated drugs be released via a diffusion mechanism. However, regarding formula C, the system exhibited the highest density of cross-links and viscosity. Hence, the release mechanism of both 5-FU and MTX follows zero-order kinetics. Moreover, it was found that *N* values for formula A were about 0.5, indicating the domination of the Fiskian mechanism, and the used drugs were released in a controlled manner. However, in the case of formula C, the value of *N* was more than 0.8, indicating that the zero-order mechanism is dominant. The results and interpretation are in good accordance with the previously reported data [45,46,47].

### 2.6. Syringeability and Injectability

The prepared hydrogels were examined for their ability to be administered intramuscularly by using 5 mm syringes. The results showed that the volume of 0.5 mL gel of formula A was easily injected and passed through the needle within 10 s upon applying a moderate finger pressing force. On the other hand, gel system C showed a higher degree of resistance to flow from the needle compared to system A. This may be due to that the viscosity of hydrogel system C is higher than that of formula A [48].

### 2.7. In Vitro Antitumor Activity and Cell Viability Studies

**Figure 4:**
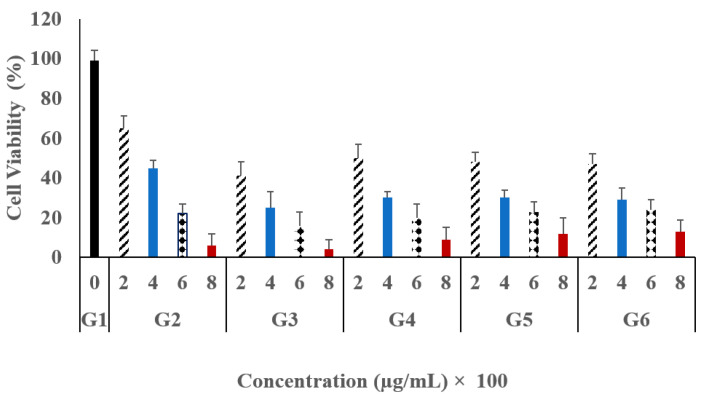
Cytotoxicity assay (% cell viability), as a function of drug concentration, loaded into the modified gel systems; G4 (8armPEG20k-CD/8armPEG20k-chol), G5 (pβ-CD/8armPEG20k-Ad), and G6 (pβ-CD/8armPEG20k-chol) compared with G2 (5-FU free saline solution) and G3 (5-FU/MTX free saline solution) against MCF-7 breast cancer cell line. The results are presented as the average of three independent measurements ± SD.

The antitumor activity of the constructed hydrogel system against MCF-7 cells was investigated using an MTT assay. In brief, the tumor cells were treated with 5-FU^®^ solution, 5-FU/MTX mixed solution, unmedicated hydrogel (used as a negative control), and the selected hydrogel systems (formulas A and C) loaded with 5-FU/MTX at different concentrations (200, 400, 600, and 800 μg/mL). MCF-7 cells were treated for 72 h. As presented in Figure 4 and Table 4 the drug-loaded hydrogel groups (hydrogel formula A) showed a significant cell viability inhibition (*p* ˂ 0.05) in comparison to the untreated group. In addition, it was noted that the dual-drug treatment (5-FU/MTX) exhibited a significant increase (*p* ˂ 0.05) in the antiproliferation capacity, compared to the single-drug treatment (cell viability of 41%, 25%, 16%, and 4%) at concentrations of 200, 400, 600, and 800 μg/mL, respectively. In contrast, the free 5-FU group showed less degree of inhibition (high cell viability) of 65, 45, 22, and 6, at the same concentrations, respectively. Noteworthy, the blank hydrogels of both formulas A and C did not show a remarkable cytotoxic effect against the normal or cancer cells. The obtained results are in good accordance with the data reported previously by our research group [34].

These results revealed that the codelivery of both drugs showed obvious synergistic antitumor activity against breast cancer cells. Moreover, the application of free drug solution (either single or multiple) exhibited a significantly higher antiproliferation activity (*p* ˂ 0.05) compared to the drug-loaded hydrogels, as illustrated in Figure 4 and Table 4.

**Figure 5:**
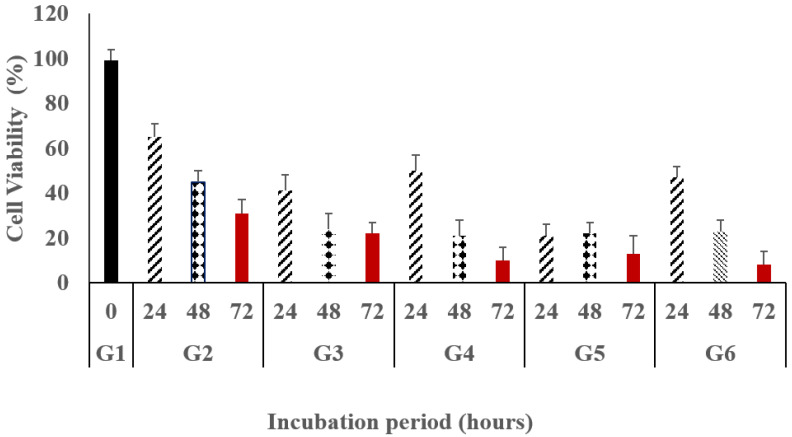
Cytotoxicity assay (% of cell viability), as a function of the incubation period, of the modified gel systems; G4 (8armPEG20k-CD/8armPEG20k-chol), G5 (pβ-CD/8armPEG20k-Ad), and G6 (pβ-CD/8armPEG20k-chol) compared with G2 (5-FU free saline solution) and G3 (5-FU/MTX free saline solution) against MCF-7 breast cancer cell line. The results are presented as the average of three independent measurements ± SD.

For example, at a concentration of 200 μg/mL, the cell viability in the case of free 5-FU/MTX solution was 41%. However, the values of cell viability for the modified loaded gels A, B, and C were 50%, 48%, and 47%, respectively. Similar results were obtained in all investigated concentrations. On the other hand, regarding the difference between the investigated gel systems, the obtained results indicated that there are no significant differences (*p* = 0.45) between them in the extent of proliferation inhibition. This may be contributed to the diluting effect of the hydrogel materials. The effect of the incubation period on the cell viability was also evaluated and the obtained result was illustrated in Figure 5 and Table 5. In this context, the data showed the cell viability after applying a concentration of 200 μg/mL for all investigated groups. Generally, all medicated groups showed a significant inhibition (*p* = 0.035) of the cell viability for the studied breast cancer cells when compared with the untreated group. Moreover, it was observed that the increase in the incubation period was accompanied by more cell viability inhibition.

The effect of the incubation period on the cell viability was also evaluated and the obtained results were illustrated in Figure 5 and Table 5. In this context, the data showed the cell viability after applying a concentration of 200 μg/mL for all investigated groups. Generally, all medicated groups showed a significant inhibition (*p* = 0.035) of the cell viability for the studied breast cancer cells when compared with the untreated group. In addition, it was observed that the increase in the incubation period was accompanied by more cell viability inhibition.

In the case of G3, the extent of cell viability was 41.3%, 24.2%, and 22.3% at 24 h, 48 h, and 72 h, respectively. However, in the case of G2, the cell viability values were 65.4%, 45.5%, and 31.4% at the same incubation periods. It was observed that all treated groups exhibited a significant antiproliferative efficacy compared to the untreated groups. By taking 24 h, as an incubation period, and 200 µg/mL as a fixed concentration, the free drug either 5-FU alone or in combination with MTX (5-FU/MTX) showed a higher impact than hydrogel systems since the cell viability in the case of free 5-FU/MTX saline solution was 41% compared with 50%, 48%, and 47% in the cases of G4, G5, and G6, respectively. Similar results were obtained for cells treated for 48 h and 72 h. Moreover, it was noted that the hydrogel systems exhibited a sustained effect compared to the free drugs. For example, at 72 h, the cell proliferation values in cases of G4, G5, and G6 were 8%, 13%, and 10%, respectively, compared to 22% in the case of the free 5-FU/MTX solution receiving group (G3).

From the obtained results, it could be concluded that the modified blank hydrogel systems showed excellent safety profiles even at high concentrations. However, in all cases, the free drug showed higher effects than the drug-loaded gels. In addition, the modified gel systems still show respectable antiproliferative effects indicating the suitability of the modified gel systems for multidrug therapy. This finding indicated that the inhibitory effect is concentration- and time-dependent. The obtained findings are in good accordance with the previously reported results [49,50].

### 2.8. Clinical Signs and Histopathological Appraisals

Remarkable improvement was observed in the clinical signs and histopathological traits of both the treated and the untreated tumor-induced groups. Notably, the drug-loaded hydrogel systems showed higher improvement patterns compared to the free-injected drug solutions. This might be attributed to the trapping efficiency of the hydrogel system, which allows the drug to remain for a longer duration of time inside the cancer tissues compared to the free drugs.

**Figure 6:**
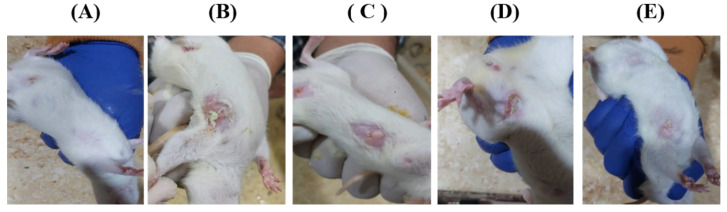
Photographs of the treated rats, including normal rat (**A**), untreated tumor-induced rat (**B**), and the various treated groups (**C**–**E**), including 5-FU/MTX saline solution injected group (**C**), the drug-loaded hydrogel system A (**D**), and the drug-loaded gel system C (**E**).

Photomicrographs of the clinical signs that appeared in both the untreated and treated groups are displayed in Figure 6. The micrographs showed a better improvement in the animals treated with hydrogel formulas A and C than those treated with the free drug solutions. These clinical signs were further supported by pathological investigations.

**Figure 7:**
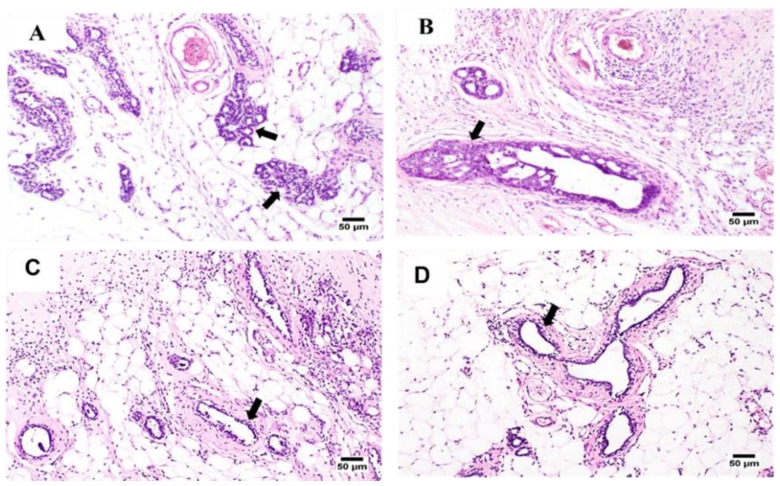
Photomicrographs display some histopathological appraisals (arrows), which were detected in the mammary gland tissues from six treatment groups: (**A**) the unmedicated group showing numerous clusters of neoplastic cells with marked edema; (**B**) the group receiving (5-FU/MTX) saline solution showing proliferating neoplastic cells with inflammatory reactions; (**C**) the group receiving hydrogel formula A showing few proliferating mammary ducts and inflammatory cells infiltration; and (**D**) the group receiving hydrogel formula C showing few neoplastic cells forming ducts with mild inflammatory edema; 100× (H&E).

Figure 7 displays the microscopic pathological traits monitored in the examined tissue sections of the tumor masses excised from the tumor-induced mammary glands. G1 samples showed marked pathological changes in the tissues of the mammary gland and its covering skin as well. Furthermore, clusters of markedly proliferated neoplastic carcinoma cells were observed and associated with dysplasia in the acini and ducts of the tumor-induced mammary gland. The neoplastic cell proliferation initiates multiple layers, papillary projections, and clusters within the glandular ducts. Malignancy in the form of cellular and nuclear pleomorphism and hyperchromasia was observed in the tumor cells.

Additionally, marked inflammatory edema, associated with intense inflammatory cell infiltration, was detected in the surrounding tissues. The skin covering the affected gland showed acanthosis with marked vacuolation in the prickle cell layer. Notably, a thick zero-cellular crust rich with intense inflammatory cells covered the glandular skin surface. Squamous cell carcinoma was detected in the skin covering some severely affected glands. In addition, a characteristic “epithelial pearl”, made up of central keratin whorl surrounded by neoplastic epithelial cells, was observed.

Mild improvement was observed in the case of G2 samples, inward projections or multiple layers lining the tumor cells were detected inside the mammary gland’s ducts, and their surrounding epithelium showed intense inflammatory cell infiltration.

Better improvement signs were noticed in the case of the G3 group, receiving the hydrogel formula A. Fewer proliferating tumor cells and inward growth or clusters of neoplastic cells were detected within the examined ducts. Occasionally, wide necrosed areas and mild-to-moderate inflammatory cell infiltration were observed.

Interestingly, the best improvement was achieved by the G4 group, which was treated with hydrogel C. The proliferating neoplastic cells were the fewest of all tumor-treated groups with a higher tendency for duct and acini formation. No tumor cell clusters or sheets were detected, and the inflammatory reaction was minimal.

### 2.9. Relative Tumor Volume Studies

**Figure 8:**
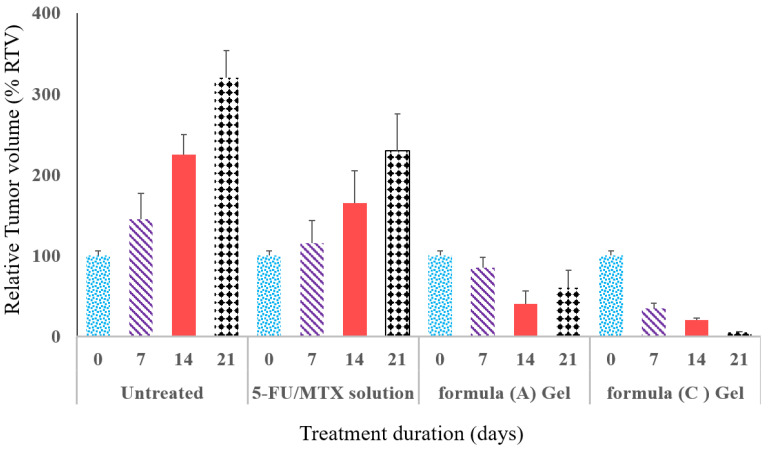
The percentage of relative tumor volume (%RTV) as an indication of antitumor efficacy of the modified hydrogel systems (formulas A and C) in comparison with the free drugs and untreated groups after their local injection into the breast tumor (*n* = 8).

The antitumor activity of the drug-loaded systems compared to the free drugs (MTX/5-FU) was evaluated by monitoring the change in the tumor volume before and after specific time intervals. After 2 months of tumor induction, the tumor will be at the size to be observed and measured by a caliper, since the volume of the tumor was about 660 ± 77.05 mm^3^. After a period of 21 days, RTV was calculated for all investigated groups and the results were illustrated in Figure 8. The results showed that there was a significant difference (*p* < 0.05) in the tumor growth between the untreated group and all other treated groups (5-FU/MTX saline solution or with 5-FU/MTX-loaded hydrogels, A and C). It was observed that 5-FU/MTX-loaded hydrogels exhibited a higher degree of tumor deduction compared with the free solution at the same concentration.

This result agrees with the above-mentioned cell viability data. The hydrogel formula C showed higher growth inhibition (8% RTV, or 92% inhibition) compared to the gel formula A (50% RTV, or 50% inhibition). In contrast, the extent of tumor inhibition was not observed in the case of free drug (220% RTV, 120% tumor growth) or untreated groups (320% RTV, or 220% tumor growth) after certain days of administration. This may be due to the short duration of action of the drug in solution compared with the modified gels. The obtained results and interpretation are in good agreement with the previously reported data [51,52].

**Figure 9:**
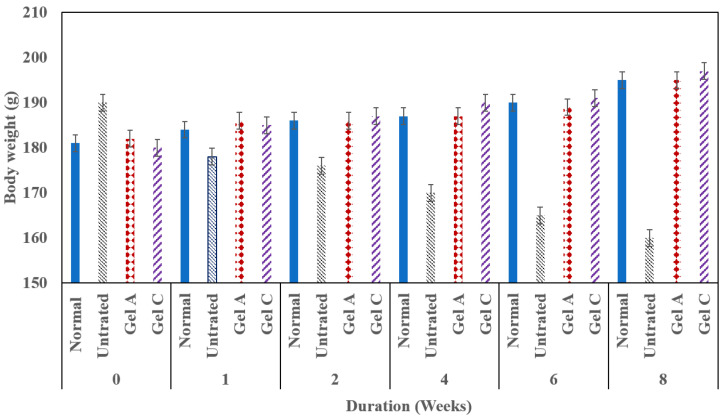
The effect of the modified hydrogel systems (A and C) loaded with dual anticancer (5-FU/MTX) on the body weight of rats in comparison with untreated animals.

The effect of medication on the body weight of the investigated rats was also studied and the results were illustrated in Figure 9. It was observed that the untreated group showed a significant weight loss compared to all treated groups. In contrast, there is no weight loss in the case of all medicated groups. This may suggest that the use of the proposed hydrogel formulations has no significant effect on body weight. In the case of group G5 (treated with hydrogel A), it was observed that after 2 weeks of treatment, the weight of animals was decreased but still higher than that of the untreated group. This may be due to the duration being only 2 weeks. While in the case of G6 (treated with hydrogel C), the duration was extended to more than 1 month.

## 3. Conclusions

In the current study, we established a straightforward procedure for the formulation of different stable hydrogel systems which are suitable for the loading and delivery of a 5-FU/MTX co-administered anti-cancer mixture. The polymers used in these hydrogel formations were synthesized successfully and characterized by using different analyses. Fortunately, the constructed hydrogel systems were stable even at high-temperature degrees since the strength and stability of the modified hydrogels increased with the increase in cross-links density. The in vitro release results showed that the quantitative release of both drugs was achieved within 3 weeks, with a release pattern depending on the composition of hydrogel systems. The SEM results showed that the modified hydrogel systems have amorphous and porous structures. The cytotoxicity studies of our modified systems indicated that the extent of cell viability inhibition was concentration- and time-dependent. Moreover, the histopathological studies showed an improvement in cancer manifestation after hydrogel injection. The best improvement was obtained in the case of group 6 (hydrogel formula D). These results confirm the potential of the modified hydrogel system as a safe and efficient drug delivery matrix for 5-FU/MTX and could be applied to other anticancer therapies.

## 4. Materials and Methods

### 4.1. Materials

Succinic anhydride (SA), cholesterol, epichlorohydrin (EP), diisopropyl azodicarboxylate (DIAD), A 1-adamantyl isocyanate, and 1-hydroxy benzotriazole (HOBt) were obtained from Sigma-Aldrich (Schnelldorf, Germany). β-Cyclodextrin (β-CD) was delivered from Wacker Chemie AG (Burghausen, Germany). Triphenylphosphine (PPh_3_), triethylamine (TEA), phthalimide, *N*-(3-dimethylaminopropyl)-*N*′-ethylcarbodiimide (EDC), sodium azide, and dibutyltindilaurate (DBDL) were supplied by Acros Organics (Geel, Belgium). Star-shaped 8-arm poly(ethylene glycol) (8armPEG20k-OH) was provided by Jenkem Technology (Beijing, China). The solvents, such as tetrahydrofuran (THF), ethanol, anhydrous dichloromethane (DCM), toluene, and diethyl ether, were of analytical grade and purchased from Merck KGaA (Darmstadt, Germany). Double-distilled water, phosphate-buffered saline (PBS), Dulbecco’s modified Eagle’s medium (DMEM), and heat-inactivated fetal bovine serum (FBS) were provided by Invitrogen GmbH (BioSera, Cholet, France). Penicillin and Streptomycin were obtained from PAA Laboratories GmbH (Tiefenbach, Austria). Noteworthy, throughout the present entire study, the glassware and vessels, utilized for trace analysis, were kept in a solution of nitric acid solution (10% *v*/*v*) for 1 day. Subsequently, they are washed several times with double distilled water. Moreover, the hazardous wastes and materials according to the classification of EPA’s and Toxic Release Inventory (TRI) chemicals list (Regulations, E.C.o.F., 2021) (Waste, I.A.L.O.H., 2021) were carefully handled as per the stated directions.

### 4.2. Methods

#### 4.2.1. Synthesis of the Host Polymers

Firstly, the polymerization of β-CD (pβ-CD) was carried out by using EP, as a cross-linking agent, in an alkaline medium and toluene according to a previously reported method [4]. The crude product was collected by precipitation from isopropanol, redissolved in distilled water, neutralized with diluted HCl, and then dialyzed against water for 6 days for further purification where fresh water was replenished every 24 h.

On the other hand, the grafted β-CD-functionalized 8armPEG polymers (8armPEG20k-β-CD) were assembled from an interaction between 8armPEG-amine [34,53] and β-CD-monoaldehyde [54] via reductive amination under the formation of secondary amine linkage (Figure 2B). In brief, the amount of 1 g of the purified 8armPEG20k-NH_2_ was allowed to react with β-CD-monoaldehyde (3.622 g, 0.4 mM, 8 eq. per each amino group) in borate buffer pH 9.4 (1 mM, 20 mL) at room temperature for 24 h. Next, the specified amount of 502 mg (1 mM, per each amino group) of sodium cyanoborohydride, as a reducing agent, was added to the reaction mixture and kept under stirring for 4 days for purification. The reaction mixture was dialyzed against water for further 3 days using a dialyzing membrane of 3.5 kDa MWCO, where fresh water was replenished every 24 h.

#### 4.2.2. Synthesis of the Polymerized Guest Molecules

Branched 8-armed poly(ethylene glycol) was functionalized by cholesterol moieties to form 8-armed poly(ethylene glycol)-cholesterol polymer (8armPEG20k-chol). The synthesis was carried out via the coupling interaction between 8armPEG-amine and cholesterol-succinate according to the procedure previously described by our group [5]. In addition, the PEG end-groups were functionalized by adamantyl moieties to give 8armPEG20k-Ad, as previously reported by Sandier et al. [55]. The preparation was carried out via the reaction of PEG with adamantane isocyanate in the presence of TEA and DBDL.

#### 4.2.3. Hydrogels’ Preparation

Initially, preliminary attempts using a different assemble composition of the hydrogels were proceeded by dissolving the prepared guest polymers (8armPEG20k-Ad or 8armPEG20k-chol) into the host-modified β-CD (pβ-CD or 8armPEG20k-CD) at low concentrations (1.0% *w*/*v*) and different ratios (*v*/*v* in a distilled water). Subsequently, the mixture solutions were frozen and lyophilized. Later, the hydrogels with the optimum concentration and ratio were prepared from the rehydration of the resulting fluffy powder by PBS, as illustrated in Table 6, formulas A–F.

#### 4.2.4. Characterization of the Prepared Hydrogels

- Visual appearance and pH of the prepared hydrogel

The prepared hydrogel systems were investigated by the naked eye for color, smoothness, 3D structure existence, transparency, and homogeneity [56]. The pH of all prepared hydrogels was detected and recorded using a digital pH meter [31].

- Rheological studies

The rheological behaviors of all the modified hydrogel systems were explored using AR 2000 cone and plate Rheometer, TA Instruments (Eschborn, Germany). The diameter of the plate was 20 mm, on which the hydrogel samples were placed with a gap size of 1 mm. The kind of a modified system (hydrogel or viscous liquid) can be determined via the investigation of both loss modulus (G″) and storage modulus (G′) as a function of three independent variables, including temperature (oscillatory temperature sweep), frequency (frequency sweep), and shear stress (stress sweep) measurements [57]. Generally, the investigated mass can be considered as a gel when the values of G′ are higher than G″ (G′ ˃ G″). In contrast, if the value of G″ is higher than G′, the system will be described as a viscous liquid and not true hydrogel.

The temperature sweep experiment was designed to investigate the temperature at which the prepared hydrogel converted to a viscous liquid (T_gel_). Practically, both G′ and G″ were monitored at a temperature range of 5–80 °C and the temperature will be gradually increased at a rate of 1 °C/min (30 s equilibrium per point) [58]. In addition, the constructed hydrogel stability and strength, both previous moduli, were determined as a function of shearing stress (μ.N.torque) because the complex shear modulus (G*) was recorded at the adjusted parameters (25 °C temperature, 1 Hz frequency, and 10 μ.N.torque) [59]. Moreover, both G′ and G″ moduli were monitored as the role of a frequency (the range of 0.1 Hz to 10 Hz) at 37 °C, since the cross-over point at which the conversion of the viscous liquid to hydrogel was investigated for the prepared hydrogels [60].

- SEM studies

The morphological characteristics of the freeze-dried hydrogels and the corresponding physical mixtures of the individual polymers were investigated by SEM. Practically, the powder samples were placed on aluminum stubs and then allowed to be coated with gold (30 mM, 8 Pa) using a fine coater for at least 10 s [61].

#### 4.2.5. In Vitro 5-FU/MTX Mixture-Loaded Hydrogels Release Study

Samples of 1 g of the prepared hydrogel loaded with both 5-FU (5 mg, 0.5%, *w*/*v*) and MTX (5 mg, 0.5%, *w*/*v*) were placed into a 3-mL glass vessel. The loaded vessels were stored in the fridge (2–8 °C) for 4 h. Then, 1.5 mL PBS was poured over the gel sample surface and allowed to be incubated in a thermostatic water bath (at 37 °C) under a shaking rate of 50 rpm. After specific intervals (each 24 h), 1 mL of the supernatant of the solution was carefully withdrawn without touching the gel mass and substituted immediately with fresh PBS to maintain the sink conditions [62,63]. Noteworthy, 5-FU/MTX mixture concentrations in each collected sample were determined spectrophotometrically at a specific maximum wavelength, using a previously developed and evaluated spectrophotometric method [42], for their simultaneous analysis. The release rate profile for each system could be constructed by plotting the calculated concentration against time. The results were presented as the average of three independent experiments ± SD.

#### 4.2.6. Kinetic Model Studies

A mechanism related to the drug release from different hydrogels was explored via analysis of the obtained release data (cumulative amounts or percentages) and allowed to be graphically plotted as a function of time using different standard kinetic model equations for fitting the data. These kinetic models are illustrated in Table 7, where *Q_t_* is the amount of 5-FU/MTX mixture released at time intervals (t), and *K* values represent the release rate proportionality which is constant and specific for each model.

Regarding the Peppas Equation, the (N) value is called the diffusion exponent. Selection of the most appropriate model was relayed on the values of (R) (correlation coefficient) which exhibits the linearity for each curve. The highest value of (R) (near unity) exhibits linearity [64]. The most fitted kinetic model can be simply detected from the highest value of (R).

**Table 7:** Kinetic models equations [65,66,67].

Model	Model	Equation No.
First order	$\log Q_{t}=\log Q_{0}-\frac{K_{1}t}{2.303}$	(1)
Zero-order	$Q_{t}=Q_{0}-K_{0}t$	(2)
Diffusion model (Higuchi)	$Q_{t}=K_{h}\sqrt{t}$	(3)
Korsmeyer–Peppas model	$\log Q_{t}=\log Kp+nlogt$	(4)

#### 4.2.7. Syringeability and Injectability

Syringeability and injectability studies were carried out by filling the syringe-needle system with the investigated samples, which were then allowed to be injected into meat pieces by finger pressure. Noteworthy, to consider the modified hydrogel systems as injectable devices, they should have a suitable consistency to pass through the needle of the syringe to the skin layers in a proper manner.

### 4.3. Ex Vivo and In Vivo Studies

#### 4.3.1. Cell Viability Studies

The antiproliferative efficacy of the modified gel systems was investigated on MCF-7 (breast cancer cell lines) using MTT assay [68]. DMEM was utilized as a cultivating medium for the cultivation of the investigated cells. For cell culture, the cells were supplemented with 10% *w*/*v* FBS and 1% *w*/*v* of both two antibiotics (100 µg/mL streptomycin + 100 I.U/mL penicillin).

The starting concentration of MCF-7 was adjusted to 1 million cells/mL. The cell medium was seeded into a 96-well plate (100 μL/each well) and then incubated at 37 °C for 24 h. Subsequently, the culture medium was replaced with 500 μL of unmedicated placebo hydrogel system A (8armPEG-chol/8armPEG-CD) (-ve control 1) and placebo hydrogel system C (8armPEG-chol/pβ-CD) (-ve control 2), 5-FU^®^ saline solution (G2), (5-FU+MTX) free saline solution (G3), (5-FU+MTX)-loaded hydrogel system (A), (5-FU+MTX)-loaded hydrogel system B (G5), and hydrogel system (C) loaded with both 5-FU and MTX (G6) at four different concentrations (200, 400, 600, and 800 μg/mL) and then they were incubated at 37 °C. For comparison, the untreated group (G1) was utilized as a positive control group.

Afterwards, 100 µL of fresh medium was added to each well, and then continue the incubation at the same temperature (37 °C) for a further 48 h. Then, 25 µL of the MTT reagent (5 mg/mL in PBS) was added to the cells and incubated for a further 4 h. Thereafter, the media was carefully withdrawn and substituted with 200 µL DMSO which was added to solubilize the formazan crystals [69]. After shaking for 5 min, the cell viability and counting were determined by measuring the optical densities using an ELISA microplate reader (Bio-Tek, Winooski, VT, USA) at 570 nm [49]. All experiments were performed in triplicate and the results were presented as the mean ± SD.

#### 4.3.2. Animal Treatment

The in vivo anticancer activity studies of both 5-FU and MTX were investigated on albino rats (weighing around 185 ± 15.1 g). The study was performed according to the ethical approval number AZ-AS/PH/2/C/2021, approved by the College of Pharmacy, Al-Azhar University. The investigated animals were obtained from the animal house at Al-Azhar University. They were acclimated at 25 °C temperature with a 12 h dark/12 h light cycle [70,71]. Forty female albino rats (breast-cancer-induced animals) were randomly allocated into five independent groups, as listed in Table 8 (*n* = 8 rats per group).

Breast cancer was induced in selected groups using DMPA (75 mg/kg) which was administered by oral gavage in 1 mL of sesame oil as previously reported [72]. Both ketamine sulfate (8 mg/kg) and xylazine (10 mg/kg) were utilized for animal anesthesia. For the comparison, a negative control group was also investigated [73]. Regarding the treatment stage, the injection of the loaded hydrogel systems was performed using a mixing syringe device (Doowon Meditec Corp., Youngin-city, Republic of Korea), while the free dug saline solution was injected directly into the tumor using traditional syringes (Doowon Meditec Corp., Youngin-city, Republic of Korea).

#### 4.3.3. Tumor Growth Measurements

After 15 days of tumor induction, it was noticed that the diameter of the tumor is about 1 cm for all rats. The size and volume of the induced tumor for all investigated rats were measured with the aid of a Vernier caliper (micrometer-Ozaki Ltd., Tokyo, Japan). The net tumor volume (TV) was calculated using Equation (5) [74,75].

(5) $$\mathrm{TV}\;({\mathrm{mm}}^{3})=1/2\times \;\mathrm{Length}\times {\mathrm{Width}}^{2}$$

After drug administration, the changes in TV (the antitumor effect) were monitored and recorded every 7 days for 21 days. The doses of the administered MTX and 5-FU were adjusted to be 40 mg/kg and 100 mg/kg, respectively. The antitumor effect was monitored by measuring the relative tumor volume (RTV), representing the tumor volume after treatment, using Equation (6) [74].

(6) $$RTV(\%)=\left[\frac{{(V}_{t})}{{(V}_{0})}\right]\times 100$$

where V_0_ is the tumor volume at 0 time (i.e., directly after drug injection) and V_t_ represents the volume at each specific time interval.

#### 4.3.4. Histopathological Appraisals and Traits

Tissue samples were taken from the tumor masses of the tumor-induced mammary glands and were fixed in formalin for 24 h. The fixed samples were processed for the usual histological procedures using H&E counterstaining of ultrathin paraffin sections (5 µm; Micro HM 360^®^ Microtome) [76]. The stained sections were examined under a light microscope (Olympus BX 46) and were digitally photographed using its connected Olympus DP 21 digital camera (Olympus Corporation, Tokyo, Japan).

### 4.4. Statistical Analysis

In the current study, the experiments were carried out three times. The presented values were the mean of these triplicate measurements ± SD. Regarding the cytotoxicity studies, the statistical significance analysis was accomplished by using one- and two-way ANOVA for multiple comparisons between the investigated groups, with a post hoc test for the paired comparison of means [77]. The difference can be considered as nonsignificant, significant, and highly significant when the probability value is *p* ˃ 0.05, *p* ˂ 0.05, and *p* ˂ 0.01, respectively.

## Figures and Tables

**Table 1:** The degree of substitution (DS), yield, and molecular weights (MWs) of the synthesized polymers utilized for the construction of hydrogel systems.

Polymer	DS (%)	Yield (%)	MW (kDa)
8armPEG20-chol	75	80–90	22.82
8armPEG20-CD	95	75–80	28.62
8armPEG20k-Ad	97	85–95	22.84
pβ-CD	65	60–62	96.00

**Table 2:** The properties of different modified hydrogel formulations.

Code	Drug Content		pH	Rheological Properties				
				T_gel_ (°C)	Cross-Over (Hz)	Stability (μ.N.Torque)	Viscosity (Pa)	Gel Strength (G*) (Pa)
	5-FU	MTX						
A	101 ± 3.1	101 ± 3.1	7.2 ± 0.2	42	2 ± 0.2	100 ± 3.2	2500 ± 100.2	3013 ± 35
B	101 ± 2.5	100 ± 1.5	7.2 ± 0.1			107 ± 4.2	92 ± 4.2	95 ± 12
C	101 ± 2.8	101 ± 2.8	7.4 ± 0.1	69	0.1 ± 0.03	1258 ± 30.2	6940 ± 70.2	6950 ± 84

**Table 3:** The kinetic models of the in vitro release studies.

		5-FU		MTX	
		Formula A	Formula C	Formula A	Formula C
Zero-order	R	0.865	0.948	0.955	0.969
	Slope	32.14	12.38	9.657	15.28
First order	R	0.853	0.963	0.918	0.974
	Slope	0.136	0.233	0.187	0.504
Higuchi diffusion	R	0.989	0.923	0.996	0.786
	Slope	25.262	36.78	38.474	45.73
Kors.–Peppas	R	0.903	0.902	0.985	0.827
	N	0.501	0.835	0.556	0.796
Best fitted model		Higuchi	Zero	Higuchi	Zero

N, the exponent of the Kors.–Peppas equation; R, the correlation coefficient.

**Table 4:** The percentage of breast cancer cell viability measurements as a function of concentration for all modified hydrogel systems compared to either free drug solutions or untreated groups.

Code	Composition	Concentration (μg/mL)				
		0	200	400	600	800
G1	Untreated group	99.4 ± 2.2				
G2	5-FU saline solution		99.5 ± 1.2	45.4 ± 3.2	22.8 ± 3.2	6.4 ± 1.2
G3	5-FU/MTX saline solution		65.4 ± 2.2	25.6 ± 2.6	16.4 ± 1.8	4.5 ± 1.6
G4	5-FU/MTX-loaded gel A		41.3 ± 3.1	30.7 ± 4.2	20.6 ± 4.1	9.6 ± 3.2
G5	5-FU/MTX-loaded gel B		50.4 ± 3.2	30.3 ± 3.9	23.7 ± 3.2	12.7 ± 4.1
G6	5-FU/MTX-loaded gel C		48.6 ± 3.0	29.2 ± 2.5	24.3 ± 2.4	13.6 ± 3.3

**Table 5:** The percentage of breast cancer cell viability measurements as a function of the incubation period for all modified hydrogel systems compared to either free drug solutions or untreated groups, taking 200 μg/mL as a fixed concentration for all groups.

Code	Composition	Cell Viability (%)/Incubation Period		
		24 h	48 h	72 h
G1	Untreated group	99.5 ± 1.2	100 ± 1.5	99.3 ± 1.1
G2	5-FU saline solution	65.4 ± 2.2	45.5 ± 1.6	31.4 ± 1.1
G3	5-FU/MTX saline solution	41.3 ± 3.1	24.2 ± 1.4	22.3 ± 1.2
G4	5-FU/MTX-loaded gel A	50.4 ± 3.2	21.3 ± 2.2	10.5 ± 1.8
G5	5-FU/MTX-loaded gel B	48.6 ± 3.0	22.8 ± 1.3	13.2 ± 1.9
G6	5-FU/MTX-loaded gel C	47.9 ± 3.2	23.5 ± 2.1	8.8 ± 2.2

**Table 6:** Composition of different hydrogel systems from equal weight (ratio 1:1 *w*/*w* ) of two inclusion complexation partners (host and guest).

Formula Code	Total Solid (%, *w*/*v*)	Polymer Composition (*w*/*w*)	
		Host	Guest
A	30	8armPEG20k-CD_7_	8armPEG20k-(chol)_7_
B	10	pβ-CD	8armPEG20k-(Ad)_8_
C	10	pβ-CD	8armPEG20k-(chol)_7_
D	10	pβ-CD	8armPEG20k-OH
E	10	β-CD	8armPEG20k-(chol)_7_

**Table 8:** Classification of the investigated animals according to the kind of medication which was taken during the study.

Code	Kind of Medication Which Was Received
Group 1	Normal nondiseased animals (Control)
Group 2	Treated with plain hydrogel (Control)
Group 3	Treated with 5-FU (100 mg/kg) + MTX (40 mg/kg) saline solution
Group 4	Treated with 5-FU/MTX-loaded hydrogel system (Formula A)
Group 5	Treated with 5-FU/MTX-loaded hydrogel system (Formula C)

## Data Availability

All data are contained within the article.
